# Cleaning and shaping the root canal with an Nd: YAG laser beam: A comparative study

**DOI:** 10.4103/0972-0707.66718

**Published:** 2010

**Authors:** Prashant P Moogi, R Nageshwar Rao

**Affiliations:** Department of Conservative Dentistry and Endodontics, K.L.E’s Institute of Dental Sciences, Bangalore – 560 022, India; 1 Department of Conservative Dentistry and Endodontics, S.D.M Dental College, Dharwad, Karnataka, India

**Keywords:** LASER Nd:YAG, Cleaning and shaping, Smear layer, Root canal

## Abstract

**Aim:**

This study was undertaken to evaluate the effectiveness of an experimental Nd:YAG laser fiberoptic delivery system compared with conventional methods for its ability to cleanse and shape the root canal space *in vitro*.

**Materials and Methods:**

Thirty-two teeth were divided into two groups. In the first group, the canals were instrumented with k files, in the second the initial preparation was done with a K file and completed with a laser beam.

**Results:**

Scanning electron microscopic evaluations showed that preparation with a laser beam is possible and results in an improvement in the cleanliness of the canal walls when compared with conventional techniques.

**Conclusion:**

Root canal preparation using Nd:YAG laser results in cleaner dentin walls when compared to conventional methods.

## INTRODUCTION

Cleaning and shaping represents a vital step in the endodontic procedure. Goals include debridement of the root canal by the removal of organic tissue and enlargement of the canal to create a suitable shape, which facilitates debridement, irrigation, and canal obturation. In order to achieve these goals, various methods have been advocated, to make the canal walls free of irregularities. Also, endodontic instruments produce organic and mineral debris and are unable to totally remove them from the canal. Chemical irrigants are recommended for use in conjunction with mechanical instrumentation in order to dissolve the debris. However, they still leave a thin coating called a smear layer on the dentinal walls and are responsible for leakage between the canal walls and the filling material and should be removed prior to the start of the filling techniques.[1]

The result of instrument cutting efficiency and ability to shape the canal is highly dependent on the design of the file and the dynamics of the instrument during the motion within the canal. Studies have demonstrated that some files cause complications during cutting or during longitudinal filing motions. It is impossible to totally remove all the debris from the canal, as frequently there remain uninstrumented areas after preparation.[1] On account of the above-mentioned reasons, there is a need to find another method to improve the quality of the canal preparation.

Lasers have certainly shown great promise in dentistry and other applications have already been studied. CO_2_ lasers have been found to harden the enamel and to reduce the effects of an acidic attack. Observations have been made, which show that the recrystallization of enamel and dentin, with modification of the crystalline structure of hydroxyapatite, is possible with a CO_2_ laser. Unfortunately, due to its thermal effect, the CO_2_ laser cannot be used for the sterilization of the dentinal surface.[1]

Nd: YAG lasers seem to have more potential applications on the hard tissue. They are effective for the prevention of dental decay.[2] In the field of endodontics, it is observed that the walls of the canals irradiated by the Nd : YAG lasers may have reduced permeability due to the melting action of the beam on the dentin.[1]

The purpose of this study was to evaluate, by an electron microscopic examination, the quality of debridement achieved by the conventional step-back technique, using files, and to compare them with the results achieved by a laser beam driven through an optical fiber.

## MATERIALS AND METHODS

Thirty-two freshly extracted, single-rooted teeth were selected and kept in a physiological solution up to the time of the experiment. Each tooth had been radiographed, in order to select specimens with canals which are patent to #15 file. Access was made, and the canals were penetrated to a working length of 1 mm less than the actual tooth length, with a #15 K-file.

The 32 teeth were divided into two experimental groups — 12 teeth were prepared in the first group, following the step-back technique and using conventional K files, and 20 teeth were prepared in the second group following the experimental technique using the laser beam.

In the first group, the teeth were instrumented using circumferential filing and the canals were flushed between each file with 3 ml of 2% sodium hypochlorite solution, with a 25-gauge needle. The preparation was performed to allow a #35 K file to reach the working length and a #60 K file to reach a level between the middle and coronal third.

In the second group, the 20 canals were instrumented with a #15 K file for 1 min to allow a # 20 file to easily reach the working length. Sodium hypochlorite irrigation was used during instrumentation. Subsequently, the fiber optic attached to the laser unit was inserted inside the canal and the length of penetration was recorded. The aim was to reach the working length of a #15 K file.

The fiber had a silica base with a diameter of 200 *μ*m, which represented the diameter of a #20 K file. This fiber was cylindrical without coating and had to move freely along the canal path. If it did not, the enlargement was repeated with a #15 K file until the fiber felt loose inside the canal.

The laser used in this study was a 35-W pulsed Nd : YAG, which produced a beam with a wave length of 1.06 *μ*m. The beam was directed through a fiber with a cooling system, which delivered a spray of air and water during exposure. The purpose of this spray was to avoid heat production in the treated areas.

During the next step, the laser was stopped and the fiber inserted just short of the working length. The laser was then activated and the enlargement was started by pressing the optical fiber against the walls, in the direction of the upper third of the canal. The fiber was used to brush the walls on the outward stroke and was replaced apically without pressure. The enlargement was performed circumferentially starting at the apical third. Then the middle and finally the coronal third were enlarged to correspond to a #60 K file at the approximate level of the juncture of the coronal and middle third.

After completion of the procedures described earlier, the teeth were split into two parts and each part was prepared for evaluation by a scanning electron microscope. The specimens were fixed with 25% glutaraldehyde and dehydrated in a graded series of alcohol from 25 – 100% each, for 10 minutes. Then they were mounted on aluminum stubs and gold sputtered.

To obtain an analysis of the observations, scanning micrographs were taken at three levels on the prepared canals: apical, middle, and coronal. The valuators were not aware of the treatment group represented by the samples. The micrographs were rated as described herewith.

Score 0 — No smear layer, open dentinal tubules

Score 1 — Moderate smear layer, Outline of dentinal tubules are observable

Score 2 — Thin smear layer covering the surface outline of the dentinal tubules

Score 3 — Heavy smear layer

## RESULTS

In this study, evaluation of the effective removal of the smear layer from the root canal was compared between the conventional method (Group I) and the Nd : YAG laser (Group II).

**Figure 1:**
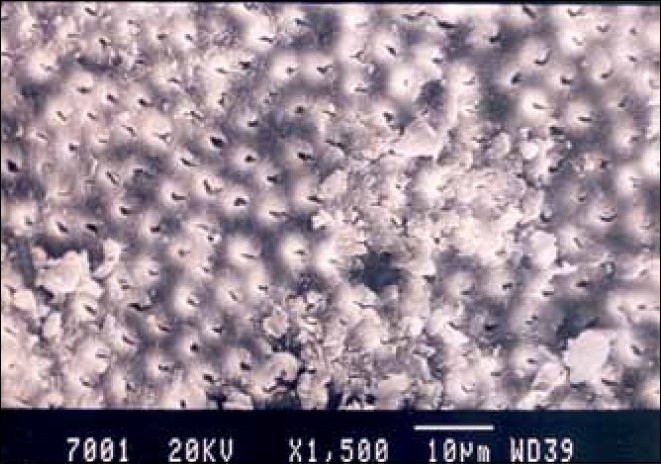
Hand-instrumented specimen apical third of the root canal

**Figure 2:**
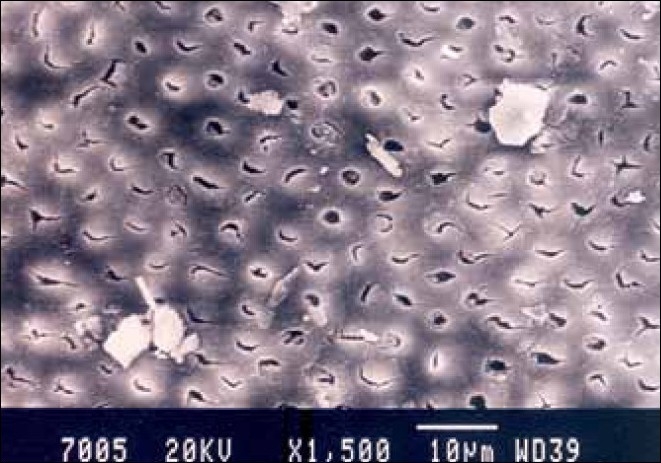
Hand-instrumented specimen cervical third of the root canal

**Figure 3:**
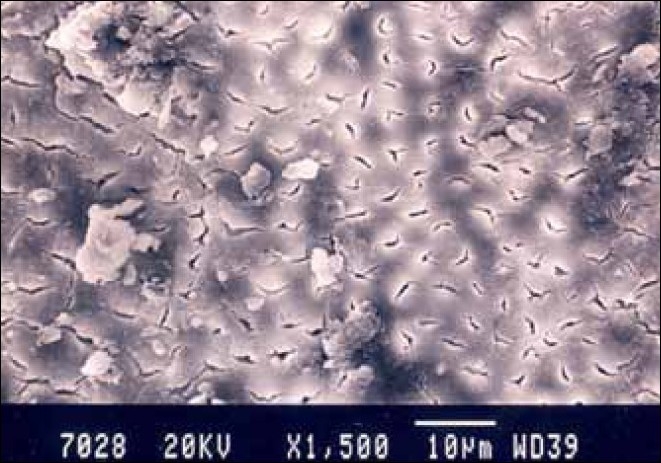
Hand-instrumented specimen middle third of the root canal

Group I specimens that were hand-instrumented with a sodium hypochlorite irrigating solution showed the presence of a smear layer in all parts of the canal. The coronal third and middle third showed the least amount of smear layer, whereas, the apical third showed the maximum amount of smear layer [Figures 1–3].

**Figure 4:**
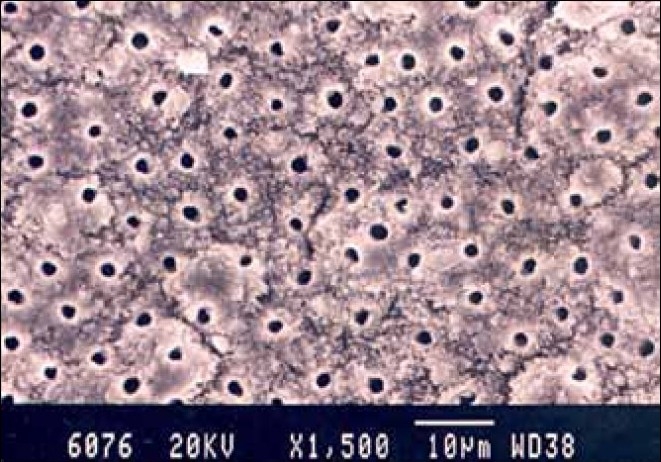
Laser-treated specimen apical third of the root canal

**Figure 5:**
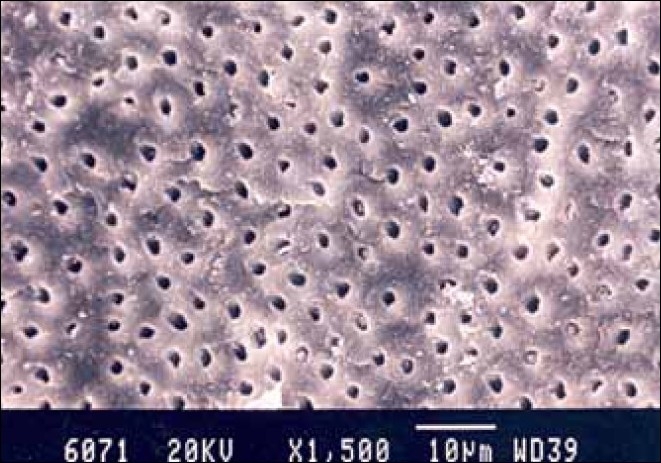
Laser-treated specimen cervical third of the root canal

**Figure 6:**
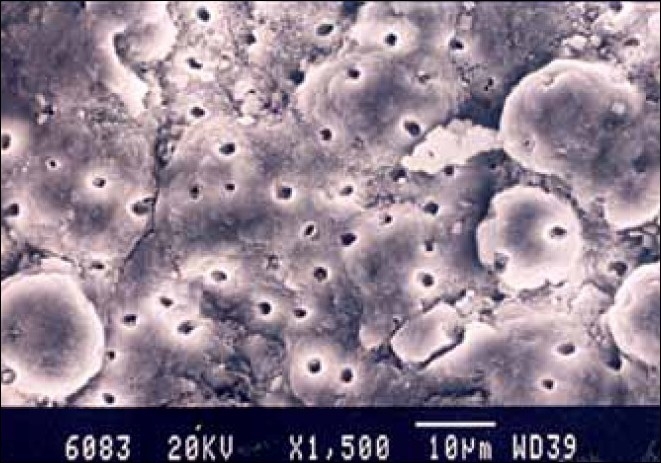
Laser-treated specimen middle third of the root canal

Group II the specimens irradiated with Nd: YAG laser showed no smear layer in the coronal and middle third. Most of the smear layer was removed and the dentinal tubules were clean and open. In the apical third a thin smear layer covering the surface outline of the dentinal tubules was present [Figures 4–6].

Results obtained by the Mann Whitney U test and Kruskal Wallis test showed a significant difference in the mean values between groups and within the groups, respectively.

At the cervical third, the Mann Whitney U test *P* (< 0.01) showed a significant difference in the mean values between Group I (2.25) and Group II (1.45). At the middle third, the Mann Whitney U test *P* (< 0.01) showed a significant difference in mean values between Group I (2.42) and Group II (1.65). At the apical third, the Mann Whitney U test *P* (< 0.01) showed a significant difference in the mean values between Group I (2.83) and Group II (2.4) [Table 1].

**Table 1:** Comparison of mean score of smear layer removed in Group I and II in different sites by (Mann – Whitney U test)

Sites	Groups	Mean	SD	U value	*P* value	Sign.
Cervical	I	2.25	0.4523	42.00	<0.01	
	II	1.45	0.6048			S
Middle	I	2.42	0.5149	78.00	<0.01	
	II	1.65	0.7452			S
Apex	I	2.83	0.3893	168.00	>0.05	NS
	II	2.4	0.6806		<0.10	S

Sign. = Significance

The Kruskal Wallis test for Group I showed a statistically significant difference between the smear layer values at the cervical, middle, and apical thirds *P* (< 0.05) K-W value → 6.3243. The Kruskal Wallis test for Group II showed a significant difference between the smear layer values at the cervical, middle, and apical thirds *P* (< 0.01) K-W value → 14.0157 [Table 2].

**Table 2:** Comparison of mean score of smear layer removed in Group I and II in different sites by (Kruskal-Wallis test)

Sites	Sum rank	K-W value	*P* value	Significance
Group I				
Cervical	276	6.3243	<0.05	S
Middle	240			
Apex	150			
Group II				
Cervical	768	14.0157	<0.01	HS
Middle	686			
Apex	376			

## DISCUSSION

One of the basic aims of root canal therapy is to clean the root canal as thoroughly as possible, to eliminate tissue debris and microorganisms.

Studies have shown that chemomechanical instrumentation, in conjunction with irrigation, is unable to totally remove the debris from the root canal walls. Ultrasonic instrumentation improves the degree of cleanliness, but it may still leave a thin coating of debris, known as a smear layer, on the dentinal walls. This smear layer is responsible for leakage between the canal walls and the filling material.[1]

An attempt to apply new technologies in conjunction with conventional treatment may increase the clinicians’ ability to render a root canal system to minimize microorganisms and debris.[1]

Recently the use of lasers has been suggested for this purpose. Hence this *in-vitro* study has been performed, to compare and evaluate the cleaning and shaping of the root canals by the pulsed Nd : YAG laser and conventional methods.[3–9]

This study included two groups, based on the method used to clean the root canal. The teeth in group I were cleaned following the step-back technique, using the conventional K-files, and the teeth in Group II were prepared by using the pulsed Nd: YAG laser, through the fiberoptic delivery system.

Our study demonstrated that the specimens that were hand-instrumented and irrigated with sodium hypochlorite solution showed the presence of a smear layer in the whole length of the root canal. In contrast the specimens irradiated with Nd: YAG laser did not have any smear layer in the cervical and middle third of the root canal. Only a thin smear layer-like substance covered the surface outline of the dentinal tubules in the apical third of the root canal. The result of this study was in accordance with the following studies.[10–14]

These results may be attributed to the manner in which the laser cuts dentin. When focused to a small spot, a pulsed Nd: YAG laser beam at a very short duration and high-peak power level can break down tissues due to the formation of plasma. Plasma formation occurs due to the ionization of the molecules. As a plasma effect is reached during the cleaning there is a transformation of dentin into ionized gas, which leaves no debris on the surface of the walls.[1]

We also observed that there was a closure of tubules caused by melting dentin in the apical third lased dentin, which showed melting, solidification, and recrystallization of the hard tissues. The structural changes did not appear to be uniform and the melted area appeared connected by areas that looked similar to a dentinal smear layer and similar to those of the non-lased specimens. This factor could reduce the permeability of the dentin walls and improve the seal after canal obturation.

The melting effect of the dentin walls could also be a means to isolate bacteria and the organic contents of the tubuli, in order to avoid their proliferation throughout the main canal.[15]

As it was an *in-vitro* study, the effect of the Nd : YAG laser on the periapical tissue was not evaluated. Our study could only identify the nature of the substance covering the dentinal tubules in the apical third of laser-irradiated teeth. Studies in the future may be directed toward identification of all these substances and evaluation of the change in permeability occurring after laser therapy.

## CONCLUSION

Within the limitations of the study, it can be inferred that canal preparation with the laser beam is possible and results in an improvement in the cleanliness of the canal walls when compared to conventional methods. From this perspective, it is important that the use of lasers should provide adequate cleaning and shaping, which contribute to the success of root canal treatment.
